# Development of an Integrated CE-Cell-SELEX Strategy for Rational Selection of Human Bone Microvascular Endothelial Cell Aptamers

**DOI:** 10.3390/molecules31111881

**Published:** 2026-05-31

**Authors:** Xinyu Fang, Wenting Pan, Jiefang Sun, Xiaojing Ding, Bing Shao, Jie Ouyang, Yiyang Dong

**Affiliations:** 1 College of Life Science and Technology, Beijing University of Chemical Technology, Beijing 100029, China; 2025430125@buct.edu.cn (X.F.); 2020201123@buct.edu.cn (W.P.); 2 Beijing Research Center for Preventive Medicine, Beijing Center for Disease Prevention and Control, Beijing 100013, China; sunjiefang@bjcdc.org (J.S.); dingxiaojing@bjcdc.org (X.D.); shaobing@bjcdc.org (B.S.); 3 College of Energy Environment and Safety Engineering & College of Carbon Metrology, China Jiliang University, Hangzhou 310018, China; ouyangjie@cjlu.edu.cn

**Keywords:** aptamer, bone microvascular endothelial cells, CE-Cell-SELEX

## Abstract

Capillary electrophoresis (CE) has proven to be an effective technique for aptamer selection. Here, we directly integrated the separation advantages of CE into a live-cell system, thereby establishing an integrated and highly efficient CE-Cell-SELEX screening model for bone microvascular endothelial cells (BMECs) without the need for negative selection. The selection progress was monitored through quantitative real-time fluorescence PCR (qRT-PCR) analysis, which yielded 7 candidate sequences from the amplified library after four rounds of selection. Flow cytometry analysis demonstrated that aptamer T-24 exhibited high affinity for BMECs, with a Kd of 111.86 ± 18.36 nM. Owing to its high affinity and specificity, coupled with its small molecular weight and non-immunogenicity, T-24 holds great potential as a biological probe for the identification and isolation of BMECs. Furthermore, molecular docking was performed by MOE 2022 software to validate the candidate sequences and assist in the identification process. The CE-Cell-SELEX method eliminates the need for negative screening and traditional elution, greatly reduces the screening cycle, and may provide a valuable reference system for the early diagnosis and precise treatment of femoral head ischemia.

## 1. Introduction

BMECs, which line the inner surface of bone microvessels, are essential for maintaining skeletal microcirculation and bone microenvironment homeostasis [1,2]. Following the 2003 outbreak of severe acute respiratory syndrome (SARS), the high incidence of steroid-associated osteonecrosis of the femoral head brought increasing attention to the pathogenic role of BMEC dysfunction [3,4]. Beyond regulating nutrient transport and metabolic waste clearance, BMECs play essential roles in angiogenesis, bone remodeling, and maintenance of the intraosseous microenvironment. Dysfunction of BMECs can impair intraosseous microcirculation and induce local ischemia–hypoxia, which is considered a critical early event in the pathogenesis of nontraumatic osteonecrosis of the femoral head (NONFH), ultimately leading to osteocyte death and structural collapse of the femoral head [5,6]. Given the critical involvement of BMEC injury in NONFH progression, interventions targeting BMEC dysfunction have attracted increasing interest as a potential therapeutic strategy. This is especially relevant for patients exposed to corticosteroids during severe coronavirus infections, as historical SARS cohorts demonstrated elevated osteonecrosis risk, raising similar concerns for COVID-19 survivors [7,8,9]. Therefore, the development of molecular ligands capable of selectively recognizing BMECs may provide new opportunities for early diagnosis and targeted intervention of NONFH.

Aptamers are single-stranded DNA (ssDNA) or RNA oligonucleotides isolated from synthetic random libraries using Systematic Evolution of Ligands by Exponential Enrichment (SELEX) [10]. Since Tuerk et al. introduced this in vitro screening and amplification technique in 1990 [11], aptamers have attracted widespread attention. These single-stranded molecules form stable secondary structures through intramolecular base pairing. Driven by non-covalent interactions like hydrogen bonding, hydrophobic effects, and van der Waals forces, they undergo target-induced conformational changes to form highly ordered 3D architectures, including stem-loops, hairpins, pseudoknots, and G-quadruplexes [12,13,14]. These distinct structural motifs precisely match the topology of their targets, yielding stable target–aptamer complexes. The binding spectrum of aptamers is incredibly broad, encompassing small molecules like metal ions and peptides, to complex entities like proteins, viruses, and whole cells [15,16]. Driven by their remarkable affinity and specificity, aptamers have recently become invaluable tools across various fields, emerging as highly effective agents in targeted therapy, biomarker detection, and diagnostics [17].

To obtain aptamers with high affinity and specificity, various SELEX-based strategies have been developed and continuously optimized. Among its variants, Cell-SELEX, first proposed by Shangguan et al. in 2006 [18], utilizes intact living cells as selection targets, enabling the simultaneous identification of multiple aptamers recognizing distinct membrane receptors under physiological conditions [19]. This strategy allows aptamers to directly target cell surface molecules without prior knowledge of specific biomarkers, making it particularly valuable for cell identification and binding studies. However, conventional Cell-SELEX has several intrinsic limitations, particularly in the separation step, which often relies on low-efficiency methods and typically requires more than 10–20 rounds of iterative selection [20,21]. This results in a time-consuming process and frequently necessitates negative selection to eliminate nonspecific binders. In contrast, CE-SELEX has emerged as a highly efficient alternative due to its ability to achieve high-resolution separation of target–aptamer complexes and free oligonucleotides in free solution without the use of immobilization media [22]. By exploiting differences in charge-to-mass ratios under an electric field, CE-SELEX enables rapid and precise discrimination between bound and unbound species, significantly improving selection efficiency and reducing the number of required rounds to as few as 2–4 cycles [23,24].

Despite the robust development of CE-SELEX, its application to complex cellular targets remains limited. Currently, the vast majority of cell-based aptamer screening is intensely focused on tumor cells [25]. Owing to their high tolerance, rapid proliferation, and minimal intergenerational expression variations, tumor cells serve as ideal and manageable screening targets. In contrast, normal human somatic cells typically exhibit long growth cycles and are challenging to culture in vitro [26], leading to their rare emphasis in screening research. Furthermore, the targets in most reported CE-SELEX studies are predominantly proteins or small molecules, with extremely few reports utilizing CE to screen specific aptamers against intact living cells [27].

In the current work, the high-resolution separation advantages of CE were integrated into a live-cell system to establish the CE-Cell-SELEX method, enabling the highly efficient screening of specific aptamers against BMECs. Four rounds of CE-Cell-SELEX were performed, and the selection progress was dynamically monitored via qRT-PCR amplification and melting curves. The final enriched pool was subsequently subjected to high-throughput sequencing on the Illumina MiSeq platform. Through sequence homology analysis and molecular docking simulations, four candidates (T-9, T-10, T-24, and T-29) were selected from the highly heterogeneous library based on their occurrences. Flow cytometry analysis demonstrated their strong binding, with candidate T-24 presenting the highest affinity toward BMECs at a nanomolar magnitude (Kd of 111.86 ± 18.36 nM). Taken together, these findings validate the effectiveness of the CE-Cell-SELEX approach in rapidly isolating high-affinity aptamers from stochastic libraries. The identification of T-24 with nanomolar affinity further underscores the capability of this strategy to generate functionally relevant molecular probes for BMEC targeting. Our work introduces a comprehensive and highly efficient process for BMEC aptamer selection, holding great promise for advancing diagnostics and therapeutics in ischemic necrosis of the femoral head.

## 2. Results

### 2.1. Schematic of Aptamer Selection and Identification

**Figure 1:**
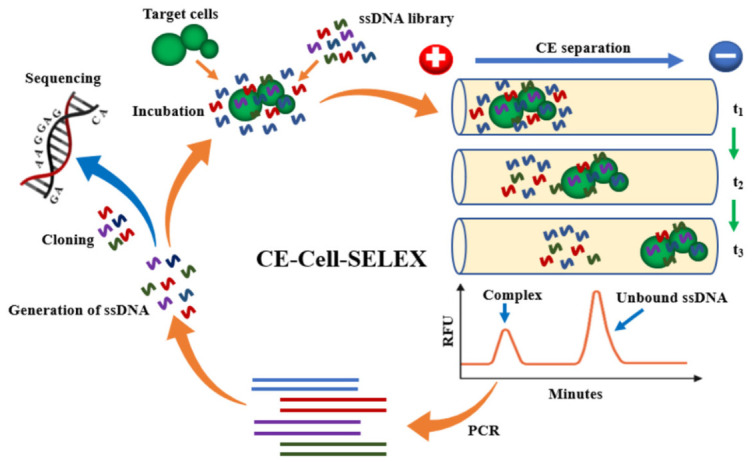
Schematic illustration of the CE-Cell-SELEX process.

Figure 1 demonstrates the specific process of aptamer selection based on CE-Cell-SELEX, as well as affinity monitoring and candidate identification. Initially, the randomized ssDNA library was incubated with the target cells in a free solution. Subsequently, the resulting incubated mixture was injected into the CE capillary for separation under high voltage. Under the applied electric field, the target cell-bound ssDNA sequences exhibited a remarkably different apparent mobility compared to the unbound ssDNA sequences due to their distinct charge-to-mass ratios. As a result, they separated into different fractions during migration. As illustrated in the electropherogram, the target cell/ssDNA complex and the unbound ssDNA successively passed through the detection window, appearing as distinct, well-separated peaks (with the complex eluting earlier). The target cell/ssDNA complex fraction was selectively collected in a vial and subjected to PCR amplification. The amplified products were then purified and utilized to generate a new, enriched ssDNA pool for the next selection round. During the iterative selection rounds, the screening progress was closely monitored through flow cytometry analysis. Meanwhile, the pools were subjected to high-throughput sequencing to analyze the enriched sequences. Furthermore, the final selection of candidate aptamers with high binding affinity towards the target cells was achieved by combining these results with molecular docking simulation experiments.

### 2.2. Feasibility Analysis

**Figure 2:**
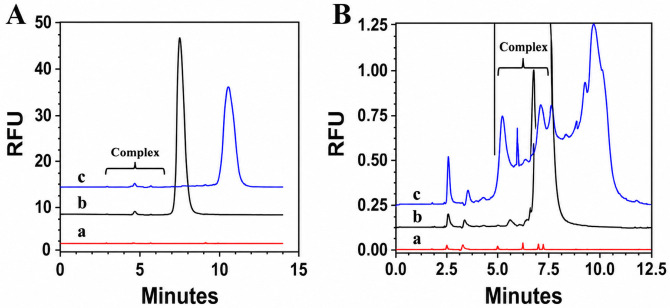
Capillary electropherograms demonstrating the feasibility of CE-Cell-SELEX for BMECs. (**A**) Full-scale and (**B**) enlarged view of the complex peak region. a: Target BMECs (2.5 × 10^6^ cells/mL); b: ssDNA library (125 nM); c: Mixture of ssDNA library and BMECs.

As shown in Figure 2, under the CE-LIF mode (excitation at 488 nm; emission at 520 nm), BMECs exhibited no characteristic peaks, indicating a lack of autofluorescence under these conditions. Conversely, the pure ssDNA library displayed a sharp, well-resolved peak at approximately 7 min. Upon incubating the library (125 nM) with BMECs (2.5 × 10^6^ cells/mL), a distinct cluster of minor peaks emerged between 4.0 and 7.5 min. Given the diverse repertoire of receptors on the cell surface, this entire region can be attributed to the BMEC-ssDNA complexes. Furthermore, compared to the pure library, the peak area of the unbound ssDNA in the mixture was significantly reduced, and its migration time was slightly delayed. This delay is presumably due to the increased cellular content causing minor capillary adsorption, thereby retarding the unbound library.

According to CE principles, substances with different charge-to-mass ratios produce different apparent mobilities. Because the DNA molecule contains phosphate residues, it is negatively charged and naturally tends to migrate toward the positive pole. Furthermore, there are a large number of proteins on the cell surface, most of which are negatively charged in a neutral environment (pH 7.4). Once ssDNA binds to cell surface proteins, the resulting complex exhibits a substantially increased molecular mass and a reduced charge-to-mass ratio compared with free ssDNA. In an uncoated fused-silica capillary, negatively charged ssDNA migrates toward the anode under the electric field, whereas the electroosmotic flow (EOF) is directed toward the cathode. Because the EOF is dominant under these conditions, the apparent migration behavior is determined by the combined effects of EOF and electrophoretic mobility. The ssDNA–protein complex possesses lower electrophoretic mobility against the EOF than free ssDNA and is therefore more readily transported by the EOF toward the detection window. Consequently, the complex reaches the detector earlier and exhibits a shorter migration time than the free ssDNA library. This behavior is consistent with the results shown in Figure 2 and preliminarily demonstrates the feasibility of screening BMEC-targeting nucleic acid aptamers under fluorescence detection mode.

### 2.3. Optimization of Binding Conditions

To investigate the effect of incubation time on complex formation and to determine the optimal duration, a time-course experiment was conducted. Equal volumes (150 μL) of a 250 nM ssDNA library and a 5 × 10^6^ cells/mL BMEC suspension were thoroughly mixed, yielding a final concentration of 125 nM library and 2.5 × 10^6^ cells/mL. Aliquots of 50 μL were sequentially sampled from this mixture at defined incubation intervals: 0, 20, 40, 60, and 90 min.

**Figure 3:**
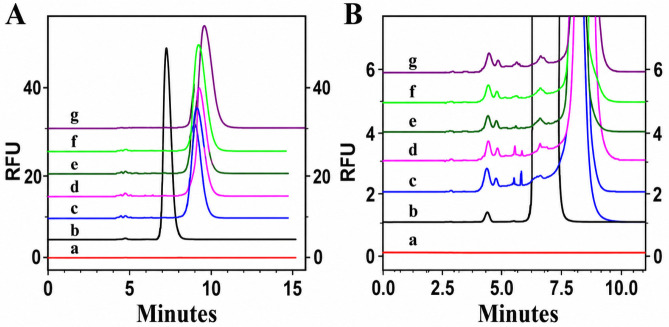
Influence of incubation time on target–aptamer complex formation. (**A**) Full-scale and (**B**) magnified capillary electropherograms. a: BMECs (2.5 × 10^6^ cells/mL); b: ssDNA library (125 nM); c–g: Target-library mixtures incubated for 0, 20, 40, 60, and 90 min, respectively.

As illustrated in Figure 3, the complex peaks did not exhibit a distinct or proportional pattern with increasing incubation time. We attribute this to the dynamic nature of the incubation process, which involves continuous adsorption and desorption. Because the specific affinity interactions in this initial CE-based screening are inherently weak and sparse, it is plausible that extended incubation time does not significantly alter the net complex yield. Furthermore, the limited injection volume typical of CE might obscure minor time-dependent variations in the complex peak. Consequently, to maximize experimental efficiency while maintaining consistency, an optimal incubation time of 20 min was selected for all subsequent selection rounds.

To definitively verify that the peaks migrating between 4.0 and 7.5 min correspond to the BMECs-ssDNA complex, the effect of target cell concentration on complex formation was investigated. A fixed concentration of the ssDNA library (125 nM) was incubated for 20 min with varying concentrations of BMECs (2.5, 2.0, 1.5, 1.0, and 0.5 × 10^6^ cells/mL). Subsequently, the mixtures were subjected to CE separation.

**Figure 4:**
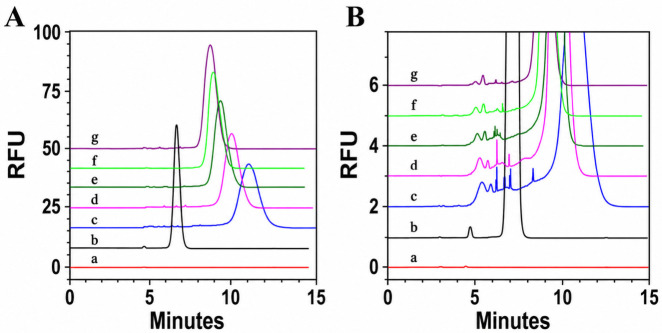
Effect of target cell concentration on BMECs-ssDNA complex formation. (**A**) Full-scale and (**B**) magnified capillary electropherograms. a: Target BMECs (2.5 × 10^6^ cells/mL); b: ssDNA library (125 nM); c–g: 125 nM ssDNA library incubated with 2.5, 2.0, 1.5, 1.0, and 0.5 × 10^6^ cells/mL BMECs, respectively.

As depicted in Figure 4, increasing the cell concentration led to a concentration-dependent enhancement of the complex peaks (Figure 4B, firmly confirming complex formation) and a concomitant decrease in the unbound ssDNA peak. Additionally, higher cell densities resulted in delayed migration times and increased peak broadening for both the complex and the free library. While prolonged separation times reduce analytical efficiency, lowering the cell dosage would unacceptably compromise the yield of the target complexes. Given that the primary objective of this study is the preparative collection of these complexes, maximizing the yield is paramount—especially considering the minute injection volumes inherent to CE. Therefore, a cell concentration of 2.5 × 10^6^ cells/mL was selected as the optimal condition to ensure sufficient complex recovery for subsequent SELEX rounds.

### 2.4. Monitoring of the Enrichment Process

**Figure 5:**
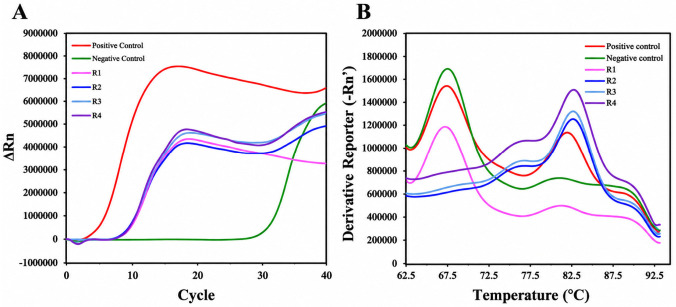
Monitoring the enrichment process via qRT-PCR. (**A**) Amplification curves and (**B**) melting curves of the secondary libraries obtained from selection rounds 1 to 4.

To account for inter-round variations in ssDNA enrichment and cell status during the CE-Cell-SELEX process, qRT-PCR was utilized at the end of each round. This allowed for the empirical determination of the optimal PCR cycle number for complex recovery and provided quality control for the collection procedure. In accordance with the methodology reported by Luo et al. [28], qRT-PCR amplification and melting curve analyses were employed to dynamically monitor the enrichment of secondary libraries from rounds 1 to 4. As shown in Figure 5A, the sigmoidal amplification curves exhibited a gradual attenuation of the initial fluorescence decline in later rounds. This phenomenon is likely associated with the progressive reduction in library heterogeneity during SELEX enrichment. Melting curve analysis (Figure 5B) provided additional evidence supporting this process. In the first selection round, the library exhibited a dominant melting peak in the 70–72 °C region. This lower-Tm population is likely associated with thermodynamically unstable heteroduplexes formed by mismatched hybridization among highly diverse DNA sequences. As SELEX progressed, the peak intensity in this region gradually decreased, while a second peak in the 82–87 °C region progressively increased. The higher-Tm population is consistent with the formation of more stable and sequence-homogeneous duplexes generated from enriched aptamer candidates. These duplexes possess fewer mismatches and therefore exhibit increased thermal stability.

The gradual transition from the lower-Tm population to the higher-Tm population suggests reduced sequence diversity and progressive enrichment of target-binding sequences throughout the CE-Cell-SELEX process. Notably, enrichment-associated melting profile changes became apparent from round 2 onward, further highlighting the high enrichment efficiency of the CE-SELEX strategy.

### 2.5. High-Throughput Sequencing and Candidate Sequence Selection

Following the confirmation of effective enrichment via qRT-PCR, the fourth-round library was sequenced via the Illumina MiSeq platform. The analysis revealed 93,581 unique sequences out of 93,621 total reads, demonstrating an exceptionally high diversity of 99.96%, and the data yielded predominantly low-frequency sequences; thus, only those with ≥2 occurrences were selected as candidates (Table S1). These candidates lacked obvious homology (likely due to inherent cell surface heterogeneity), necessitating further computational homology analysis.

Subsequent sequence homology analysis using the Clustal Omega web server (https://www.ebi.ac.uk/Tools/msa/clustalo/, accessed on 28 May 2026) (Figure S1) clustered the remaining 27 sequences into nine distinct families (Figure S2). From these, seven representative candidates together with one control sequence were selected for further analysis. Their secondary and tertiary structures were subsequently predicted using the mfold server and RNAComposer, respectively. Previous studies have demonstrated that BMECs exhibit high expression levels of CD31 and vWF, both of which are widely used for BMEC characterization and purity assessment [29]. In addition, CD34 is a well-established endothelial cell marker that exhibits strong positivity in immunostaining assays [30]. Therefore, CD31, vWF, and CD34 were selected as representative endothelial-associated proteins for preliminary molecular docking analysis using MOE software. The candidate sequences were comparatively evaluated according to their predicted minimum binding energies with these endothelial-associated markers. The predicted docking conformations of the seven candidate sequences with CD31, vWF, and CD34 are presented in Figure S3, and the corresponding minimum binding energies are summarized in Table 1.

As shown in Table 1, all seven candidate sequences exhibited more favorable predicted binding energies than the control sequence T-112. Among them, T-24 and T-29 consistently displayed relatively lower minimum binding energies toward the three endothelial-associated proteins (CD31, vWF, and CD34), suggesting potentially favorable interaction profiles in the molecular docking simulations. Therefore, these two sequences were considered promising candidate aptamers for subsequent experimental validation.

**Figure 6:**
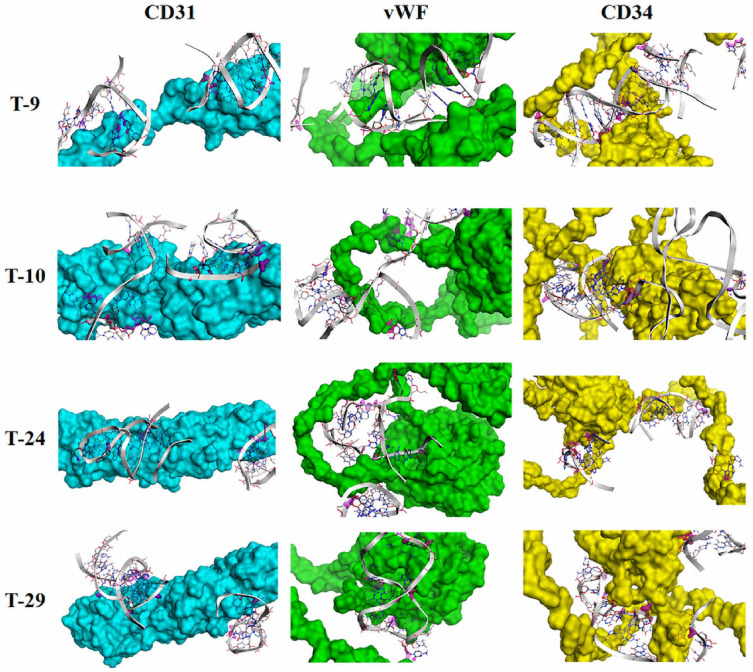
Molecular docking results of candidate sequences.

By incorporating molecular docking as a preliminary in silico evaluation step, the candidate selection process could be further refined and prioritized prior to experimental characterization. To experimentally assess the reliability of the docking predictions, four candidate sequences (T-9, T-10, T-24, and T-29) exhibiting comparatively favorable overall docking profiles were selected for subsequent in vitro binding analysis. The detailed docking interaction patterns between these four candidate aptamers and the three endothelial-associated proteins are presented in Figure 6.

### 2.6. Flow Cytometric Analysis of Binding Affinity

**Figure 7:**
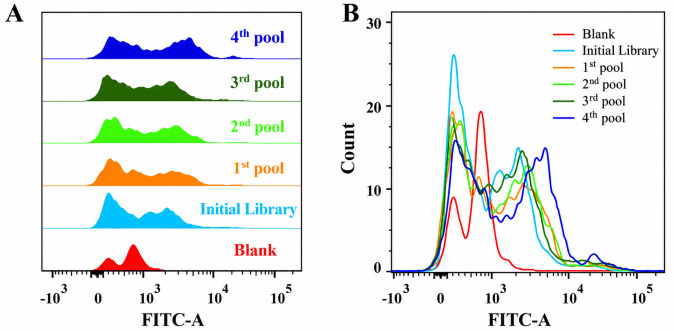
Flow cytometric analysis of library binding to BMECs. (**A**) Stacked histogram representation of fluorescence intensity distributions. (**B**) Overlay histogram representation of the same fluorescence intensity distributions.

To further validate the progressive enrichment of the secondary libraries indicated by the qRT-PCR results, flow cytometric analysis was performed using BMECs as the target cells. We evaluated the binding profiles of the FAM-labeled initial random library alongside the secondary libraries generated from selection rounds 1 to 4. As illustrated in Figure 7, the resulting fluorescence histograms display a characteristic bimodal distribution. The primary peak, situated below the 10^3^ threshold on the *x*-axis, corresponds to the intrinsic autofluorescence of the background cells. Conversely, the secondary peak on the right reflects the specific fluorescence intensity resulting from the binding of the respective libraries to the BMECs. Notably, with each successive round of selection, this specific binding peak exhibited a continuous rightward shift along the fluorescence axis. This steady enhancement in fluorescence intensity on the target cells definitively confirms the successful and progressive enrichment of the aptamer library.

**Figure 8:**
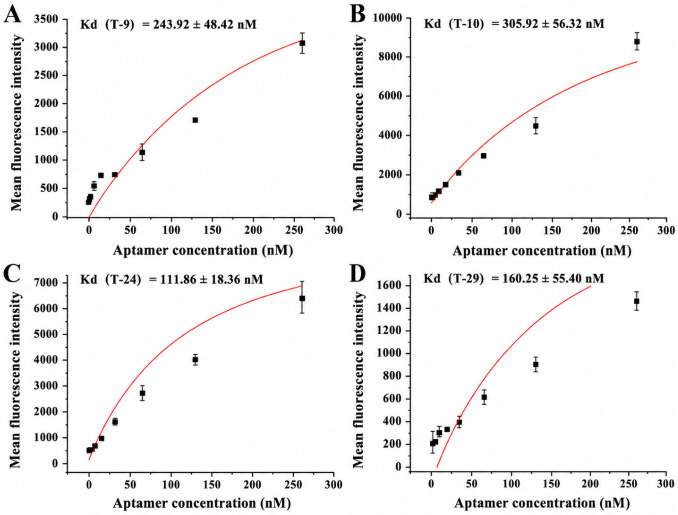
Affinity characterization of the candidate aptamers. (**A**) T-9 (**B**) T-10 (**C**) T-24 (**D**) T-29.

Based on the molecular docking simulations, the initial seven candidate sequences were narrowed down to four finalists: T-9, T-10, T-24, and T-29. To experimentally validate these computational predictions, flow cytometry was further employed to determine their binding affinities. By analyzing the mean fluorescence intensity at various concentrations, binding curves were plotted and dissociation constants (Kd) were calculated (Figure 8). All candidates demonstrated nanomolar binding affinities to BMECs, with T-24 exhibiting the lowest Kd. Crucially, the experimental affinity ranking (T-24 > T-29 > T-9 > T-10) is highly consistent with the minimum binding energies predicted by the molecular docking simulations against the biomarker vWF. These findings demonstrate that molecular simulation is a highly reliable approach for evaluating aptamer–target interactions. Consequently, utilizing docking simulations to narrow down the candidate pool is a highly feasible strategy that can significantly improve the efficiency of aptamer identification.

## 3. Materials and Methods

### 3.1. Instruments

The PA 800plus Capillary Electrophoresis system (Beckman Coulter, Fullerton, CA, USA) was used for CE experiments. The ETC811Plus Thermal Cycler (Suzhou Dongsheng Xingye Scientific Instrument Co., Ltd., Suzhou, China) was applied when performing PCR. The Thermo3110 CO_2_ incubator (Thermo Fisher Scientific, Waltham, MA, USA) was used for cell culture. The VD-650 vertical clean bench (Haier, Qingdao, China) was utilized to provide a sterile working environment. A 5424R high-speed refrigerated centrifuge (Eppendorf, Hamburg, Germany) was used for sample centrifugation and separation. The Mini-Sub horizontal electrophoresis system (Bio-Rad, Hercules, CA, USA) was applied for nucleic acid separation. Smart Gel II integrated gel imaging analysis system (Beijing Sage Creation Science Co., Ltd., Beijing, China) was used for gel imaging and fragment analysis.

### 3.2. Reagents and Materials

The 85-nt randomized ssDNA library (5′-FAM-AAG GAG CAG CGT GGA GGA TA-N45-T TAG GGT GTG TCG TCG TGG T-3′), the 5′-FAM-labeled forward primer F1 (5′-FAM-AAG GAG CAG CGT GGA GGA TA-3′), and the reverse primer R1 (5′-ACC ACG ACG ACA CAC CCT AA-3′) were synthesized by Sangon Biotechnology (Shanghai, China). These oligonucleotides were dissolved in ddH_2_O to prepare 100 μM stock solutions and stored at −20 °C. Prior to use, the library was thawed at 4 °C, diluted to the desired concentration with sample buffer, denatured at 95 °C for 5 min, and immediately snap-cooled to ensure proper conformational folding.

Na_2_B_4_O_7_·10H_2_O, H_3_BO_3_, H_3_PO_4_, NaCl, KCl, MgCl_2_, and Tris-HCl were purchased from Beijing Reagent Plant (Beijing, China). Na_2_HPO_4_ and KH_2_PO_4_ were obtained from Tianjin Jinke Chemical Industry Research Institute (Tianjin, China). Five primary buffers were prepared and used in this study: gel electrophoresis buffer: 0.5× TBE; CE running buffer (RB): 15 mM Na_2_B_4_O_7_·10H_2_O, 90 mM H_3_BO_3_, 250 mM sucrose; CE sample buffer (SB): 10 mM Na_2_B_4_O_7_·10H_2_O, 90 mM H_3_BO_3_, 176 mM sucrose; flow cytometry binding buffer (BB): 4.5 g/L D-glucose, 5 mM MgCl_2_, 0.1 mg/mL salmon sperm DNA, 1 mg/mL BSA dissolved in DPBS; and flow cytometry washing buffer (WB): 4.5 g/L D-glucose, 5 mM MgCl_2_ dissolved in DPBS. All solutions were filtered through a 0.22 μm aqueous filter membrane prior to use.

### 3.3. Cell Culture and Experimental Treatments

The orthopedic team of Director Sun Wei at the Institute of Clinical Research, China–Japan Friendship Hospital, donated primary BMECs, which were isolated from clinical bone tissue samples of orthopedic patients with osteonecrosis of the femoral head. BMECs were grown in ECM medium and stored in an incubator at 5% CO_2_ and 37 °C, with the liquid replaced every other day. Cells were cultivated in suitable volume culture flasks and screened when they reached 90% fusion cell density. Third-generation cells were employed as much as feasible in the screening procedure due to the high demand for BMECs and to avoid morphological alterations or intergenerational expression variability in bone microvascular endothelial cells.

### 3.4. CE Procedures

For the CE-Cell-SELEX process, the ssDNA library was first denatured at 95 °C for 5 min and cooled to 4 °C, followed by incubation with target BMECs in the sample buffer for 20 min at 25 °C. The resulting equilibrium mixture was plug-injected into an uncoated fused silica capillary (75 μm inner diameter, 40.2 cm total length) at 0.5 psi for 16 s. Given the total capillary length of 40.2 cm, a separation voltage of 15 kV was applied, generating an electric field of 375 V/cm, and the capillary temperature was maintained at 20 °C. The unbound ssDNA and BMEC/ssDNA complexes were monitored by a LIF detector (488 nm excitation and 520 nm emission). Based on the separation profile, the complex fraction was automatically collected into a PCR tube containing 50 μL of PCR mixture. The capillary was rinsed with running buffer for 2 min before each run, while new capillaries were initially activated with 1 M NaOH for over 10 min. The collected complex fractions were then subjected to qPCR amplification (denaturation at 95 °C for 30 s, annealing at 60 °C for 30 s, and extension at 72 °C for 30 s), with the optimal number of cycles determined by the qPCR amplification curves of the BMEC-bound sequences. Subsequently, asymmetric PCR was performed to generate an abundance of ssDNA. Finally, the amplified ssDNA was separated from the dsDNA via agarose gel electrophoresis and recovered to serve as the secondary library for the next selection round.

### 3.5. High-Throughput Sequencing and Sequence Selection

To monitor the sequence enrichment of the secondary libraries, the amplified PCR products were submitted to Sangon Biotechnology (Shanghai, China) for high-throughput sequencing on the Illumina MiSeq platform. Subsequently, sequence homology analysis of the enriched pools was performed using the Clustal Omega web server to identify representative sequences. The secondary and 3D structures of these sequences were predicted using the mfold web server and the RNAComposer processor, respectively. Furthermore, molecular docking simulations between the target protein and the candidate sequences were performed using MOE software. The final candidate aptamers were selected by comparing the minimum binding energies required for their interactions with the target protein.

### 3.6. Flow Cytometry Binding Analysis

To evaluate sequence enrichment during SELEX, FAM-labeled ssDNA pools from each selection round were incubated with BMECs (30 min, 4 °C), washed twice, and analyzed by flow cytometry. The unselected initial FAM-ssDNA library served as the blank control for background fluorescence. All experiments were performed in triplicate.

To determine their dissociation constants, BMECs were incubated with varying concentrations of the FAM-labeled aptamers at 4 °C for 30 min, after which the fluorescence intensity was measured by flow cytometry. Each concentration group was tested in triplicate, utilizing the initial library as a blank control. The mean fluorescence intensity (MFI) of each group was recorded. The Kd values of the aptamers toward BMECs were calculated by fitting the MFI of the cell–aptamer complexes against the aptamer concentrations using OriginPro 2018 software (OriginLab Corporation, Northampton, MA, USA). Non-linear regression was performed according to the equation:

(1) $$Y=\frac{B_{max}X}{K_{d}+X},$$

where *X* represents the aptamer concentration (mol/L), *Y* represents the corresponding MFI, and *B_max_* denotes the maximum fluorescence intensity.

## 4. Conclusions

In this study, CE-SELEX technology, characterized by high-resolution separation, was integrated with Cell-SELEX to explore the screening of specific targeting aptamers against BMECs. To conclude, the following findings are listed: (1) The introduced CE-Cell-SELEX method provides a highly efficient selection within only four rounds, allowing key steps to be completed in a single day while yielding high-affinity aptamers. (2) This mode achieves the targeted collection of complexes through differential migration based on varying charge-to-mass ratios. (3) The screening progress can be dynamically monitored via qRT-PCR melting curves; a continuous peak area shift from the T_m_ 70–72 °C region to the T_m_ 82–87 °C region confirms the gradual enrichment of the secondary libraries. (4) Strategic approaches, including asymmetric PCR purification, homology analysis, and flow cytometry experiments, are utilized to ensure library purity and evaluate the candidate sequences. (5) The binding abilities of candidate sequences to three target proteins (CD31, vWF, and CD34) were characterized by molecular docking simulations, which helps overcome the heterogeneity caused by multiple PCR amplifications and ensures precise selection. Therefore, CE-Cell-SELEX provides a highly efficient and targeted approach for somatic cell aptamer selection.

Moving forward, these results hold great promise for advancing aptamer-based diagnostics and therapeutics in ischemic necrosis of the femoral head, while also providing a broadly applicable CE-Cell-SELEX strategy for the rapid selection of cell-specific aptamers in diverse biomedical fields, including disease diagnosis, targeted imaging, and precision therapy.

## Figures and Tables

**Table 1:** Predicted molecular docking energies between candidate sequences and target proteins (CD31, vWF, and CD34).

Seq.	Minimum Binding Energy (kCal/mol)		
	CD31	vWF	CD34
T-1	−71.2778	−72.4742	−71.2528
T-9	−70.6930	−70.0822	−85.5562
T-10	−89.2860	−69.8530	−81.7917
T-18	−70.7950	−71.7956	−73.3679
T-21	−78.0487	−65.8643	−76.0053
T-24	−71.7326	−78.5587	−77.5027
T-29	−77.9389	−74.1082	−76.7672
T-112	−68.4419	−67.7466	−70.2021

## Data Availability

Data are contained within the article and Supplementary Materials.

## Supplementary Materials

The following supporting information can be downloaded at https://www.mdpi.com/article/10.3390/molecules31111881/s1. Table S1. Base composition of candidate sequences. Figure S1. Phylogenetic analysis of selected sequences. Figure S2. Sequence alignment and family classification of candidate aptamers. Figure S3. Molecular docking results of candidate aptamers with CD31, vWF, and CD34.
