# Supplementary material for: Development of an Integrated CE-Cell-SELEX Strategy for Rational Selection of Human Bone Microvascular Endothelial Cell Aptamers
Source: Molecules. 2026 May 31;31(11):1881. doi: 10.3390/molecules31111881 (PMC13258299; doi:10.3390/molecules31111881)
Supplement: Supplementary file 1 [file molecules-31-01881-s001.zip › molecules-4321986-supplementary.pdf]

Supplementary Materials

Results and discussion

High-Throughput Sequencing and Candidate Sequence Selection

Following the confirmation of effective enrichment via qRT-PCR, the fourth-round library was sequenced via the Illumina MiSeq platform. The analysis revealed 93581 unique sequences out of 93621 total reads, demonstrating an unexpectedly high diversity of 99.96%. Contradicting the qRT-PCR results, the data yielded predominantly low-frequency sequences; thus, only those with ≥ 2 occurrences were selected as can-didates (Table S1). These candidates lacked obvious homology (likely due to PCR-induced heterogeneity), necessitating further computational homology analysis.

Table S1. Base composition of candidate sequences.

Seq.	Sequence (5′-3′)
T-1	CGCTGGTCACTTCGGTCGTGTTCTCCGTACTCACTGGGCTGTCTG
T-2	GTCATTTTGCTGTTGGGTAATCGGTTACCGGTAGGG
T-3	TCGTCATGTGTACTGGAATGTCTGTGTGATGAGGTTCGTACTG
T-4	ACCCGCCCTAACTTTCCTCCCCCTTGCCCCAATCTCTGTACGAGG
T-5	GTGCCGTAGGCTTCGGTGTGCTTCTCATGGGCTTCAGGTAATGCC
T-6	TGCCCATCGTACCCTCCCCACCTCAGGGTATTGTCACCTTGGAAT
T-7	TCAGCCCCTTCCCCCTGTCTCGTCCTTGTCCATGGGTGTCTC
T-8	TGCCACTCTCTTCGATCCCTCCTTCCCCTAGTCCTCCCCCTCGT
T-9	ACGCGGCTGCGGTACTACCTTACTCTTTTGGTAATGGCTCAAGTG
T-10	GGCTGCGTGTACGGGTACTGGGTGTTGTTTGGGTGTGCTCTGATC
T-11	AGTGGCTACCCAGATCTGCTCAATTGACGTCCAAGTGTGTGTCCA
T-12	ACTTACTATCAACGGAGCTGTCGACGGTCCTTGCCCCCCGTTCCT
T-13	CACGTTAGATATTGACCCACATGTAGAGCGTGTCCAAAGTTACCG
T-14	CATCTTAGCTCGATTGCGGGTCCCTTATCATTGTTCTATGGCTCC
T-15	GCAAGGTCTAGCTGGCAGGATCGTTGTATGTTTTTGTCCGTACTG
T-16	CTTCCCCAACCTATACATCTATCCCCTCCTCC
T-17	TCGTTAGTGTTGTCGGGTTCCAGGGGTTTGGCTTCGTGTTGCGT
T-18	CTCCATGCACAGGTGCGATCGTGCCCCGGTATTTCCCTTTCCTCC
T-19	TCCCCTTGTCCCCCATTCTACTCAGTCCGATC
T-20	GCCCGCTCACCTTGTCGTGTTAGTTCATTTGCTTCGGTTATGCGG
T-21	CACCTCGACAAACAGCTATCGTAACCTCCCCCTCCCTCCTCCTCC
T-22	TCGAAGTCTTCCCGTTTGTGTACCCCCCTCGCCCCCTCCCAGTCC
T-23	GGTGTGATTTGGTTGCCATTCGGTCAGTTCGGTCTGTGGTGGGCC
T-24	GTCCATTGCCGTGTCTATTTGTTGGGTTCGTTTCCTGGCTCTCCG
T-25	TGTCGGTGTTACGGGTTTCGTGTTCAGGGTCGGAGGGTGTGCATG
T-26	GAATCCCGTCCTGTTTCGCTTTAATAACTGTATCTTGCCGTACCG
T-27	GTATCACAATCTGTCAACCCCAACTCGCTATTGGTTCGCTCTACCG
T-28	TGACCTCACCCGTTTTGTCGCATGTACGGCTTCCTCCTCTCGTTC
T-29	AACTCGTCCCTTCCCACTCCCTTTTCCCCCACCACGTCCCTTCCC

Table S1. Base composition of candidate sequences.

Seq.	Sequence (5′-3′)
T-30	TTGTGGCCAAATACCTTGCGGGGGGTTCTCCG
T-31	TACGGGCCCCCCTGTCGAAAGCTCTCGGTTTCGATCCTTCGTACT
T-32	CGATGCTACGGACCAGATACTTCCCCCTTACCCTCTACCCCTCTG
T-33	ATTGGGCCTACGTCATACCTGTCATTGACTCCCGTGCAGATTCCT
T-34	TCCCCCTCCATTCATTGGCAGCGTGAATCCGTAATCCCTGTACCG
T-35	GTTGCCGATGGCCGAGCTCTAGTCTTCTTGCTTCGTG
T-36	ATCCATTGTCGTGTCCTACGAGTTCCAGTGCTCTGTGTTGTTGGT
T-37	CAAGTCTCTGAAACATCCGGGTCTCTGTCTTCAACTGCGCTTGT
T-38	TGCGTTACATTTTCTTGTGTGAGCCCCTGCTACCGGAAGG
T-39	CCGGAAGGAAGGTTTGTCCTGTCCCCGTGTTGGGCCCTCTGTGCC
T-40	TTGTCCCAACCTCTCCCACTTCCCTTTCCGTCTCCCTGTTTACCCA

Subsequent sequence homology analysis using the Clustal Omega web server (Figure S1) clustered the remaining 27 sequences into nine distinct families (Figure S2). In Figure S2, candidate sequences within each family are highlighted in yellow; conserved regions are indicated in red and green, while sequences exhibiting partial similarity are shown in blue and orange. From these, seven representative candidates along with one control sequence were selected for further analysis. Their secondary and tertiary structures were subsequently predicted using the mfold server and RNAComposer, respectively.

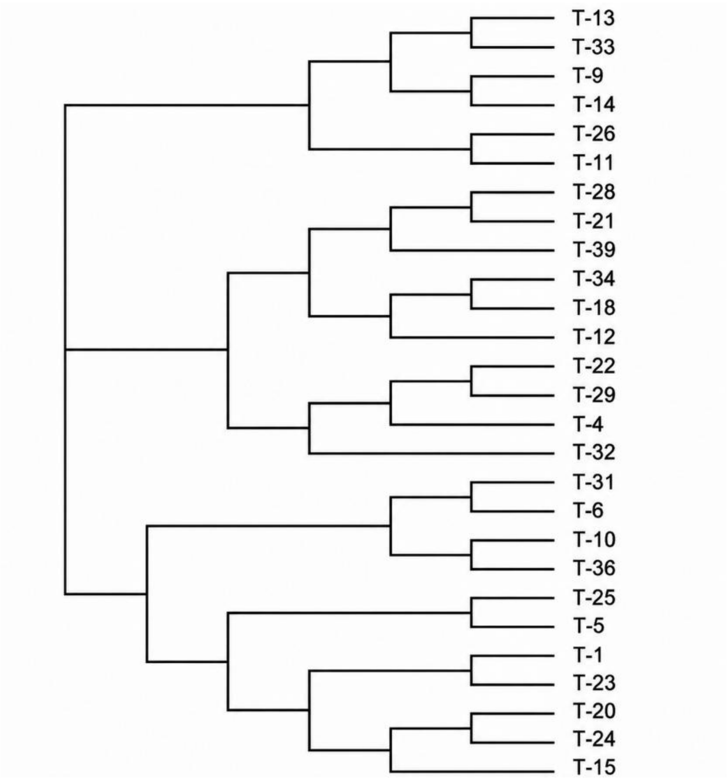

Figure S1. Phylogenetic analysis of selected sequences.

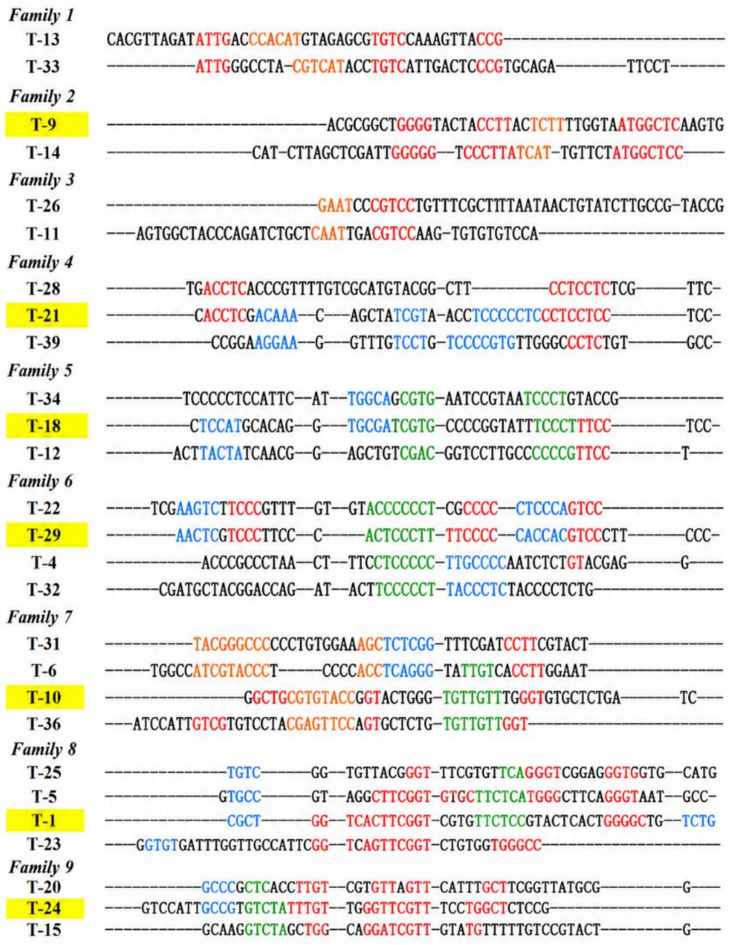

Figure S2. Sequence alignment and family classification of candidate aptamers.

Our previous studies have demonstrated that BMECs exhibit high expression levels of both CD31 and vWF. Our previous studies have demonstrated that BMECs exhibit high expression levels of both CD31 and vWF. Accordingly, these two markers are routinely employed to assess BMEC purity, a practice widely accepted in the field. In addition, CD34 is a well-established endothelial cell marker that exhibits strong positivity in immunostaining assays. Therefore, CD31, vWF, and CD34 were selected as target proteins for molecular docking simulations using MOE software. The candidate aptamers were evaluated by comparing their minimum binding energies toward these targets. The predicted docking conformations of the seven candidate sequences with CD31, vWF, and CD34 are presented in Figure S3.

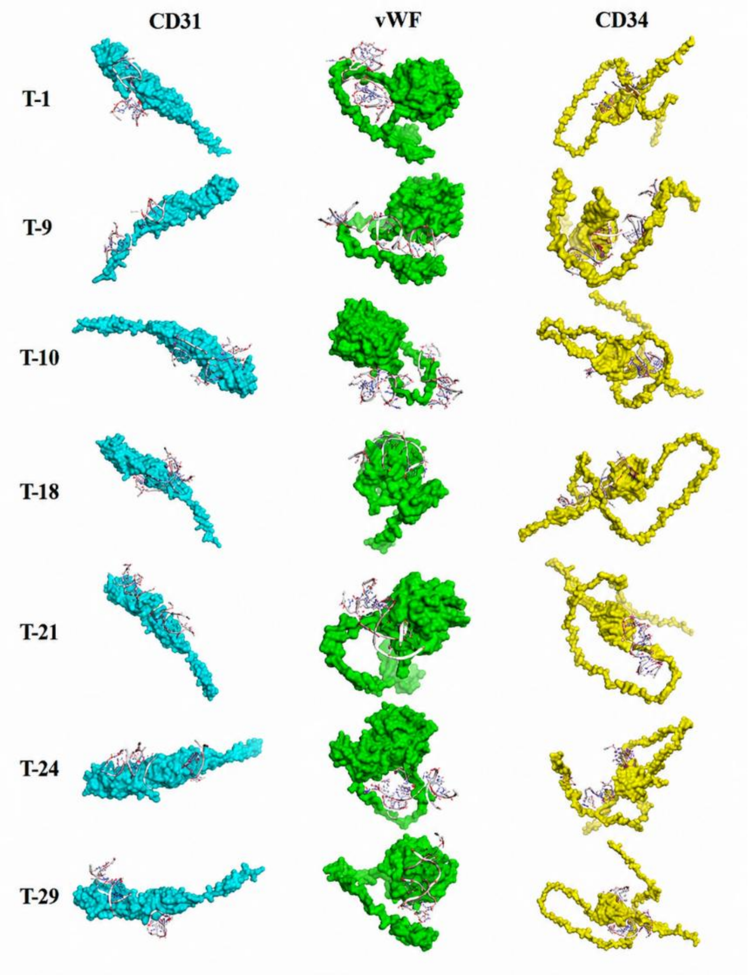

Figure S3. Molecular docking results of candidate aptamers with CD31, vWF, and CD34.
